# Implementing a digital patient feedback system: an analysis using normalisation process theory

**DOI:** 10.1186/s12913-020-05234-1

**Published:** 2020-05-07

**Authors:** Bie Nio Ong, Damian Hodgson, Nicola Small, Papreen Nahar, Caroline Sanders

**Affiliations:** 1 NIHR School for Primary Care Research, University of Manchester, Manchester, UK; 2 Sheffield University Management School, University of Sheffield, Sheffield, UK; 3 Brighton and Sussex Medical School, Department of Global Health and Infection, Brighton and Sussex Medical School, University of Sussex, Brighton, UK

**Keywords:** Digital methods, Patient feedback, Primary care, Acute care, Mental health, Normalisation process theory

## Abstract

**Background:**

Patient feedback in the English NHS is now widespread and digital methods are increasingly used. Adoption of digital methods depends on socio-technical and contextual factors, alongside human agency and lived experience. Moreover, the introduction of these methods may be perceived as disruptive of organisational and clinical routines. The focus of this paper is on the implementation of a particular digital feedback intervention that was co-designed with health professionals and patients (the DEPEND study).

**Methods:**

The digital feedback intervention was conceptualised as a complex intervention and thus the study focused on the contexts within which it operated, and how the different participants made sense of the intervention and engaged with it (or not). Four health care sites were studied: an acute setting, a mental health setting, and two general practices. Qualitative data was collected through interviews and focus groups with professionals, patients and carers. In total 51 staff, 24 patients and 8 carers were included. Forty-two observations of the use of the digital feedback system were carried out in the four settings. Data analysis was based on modified grounded theory and Normalisation Process Theory (NPT) formed the conceptual framework.

**Results:**

Digital feedback made sense to health care staff as it was seen as attractive, fast to complete and easier to analyse. Patients had a range of views depending on their familiarity with the digital world. Patients mentioned barriers such as kiosk not being visible, privacy, lack of digital know-how, technical hitches with the touchscreen. Collective action in maintaining participation again differed between sites because of workload pressure, perceptions of roles and responsibilities; and in the mental health site major organisational change was taking place. For mental health service users, their relationship with staff and their own health status determined their digital use.

**Conclusion:**

The potential of digital feedback was recognised but implementation should take local contexts, different patient groups and organisational leadership into account. Patient involvement in change and adaptation of the intervention was important in enhancing the embedding of digital methods in routine feedback. NPT allowed for a in-depth understanding of actions and interactions of both staff and patients.

## Background

In the last decade, the value of patient feedback on health services has become widely accepted. In the United Kingdom (UK), the collection of patient experience data has routinely been collected via the NHS patient survey programme by the Picker Institute for the Care Quality Commission (Picker Insitute Europe). Furthermore, annual surveys of patient experience are carried out in specific areas such as the national GP Patient Survey (1.3 million patients) [1] and the national inpatient survey (64,000 patients) (http://www.nhssurveys.org/surveys). These are retrospective postal surveys with respectable response rates, commonly between 30 and 40%. In 2012 the Friends and Family Test (FFT) was introduced in the English NHS, which asks whether patients would recommend the service to friends and family as a means of gathering simple and timely patient experience feedback [2]. Its emphasis on near ‘real time’ feedback has been highlighted in a number of key reports [3, 4] as a contribution to enabling safe care, and this points to the limitations of traditional pen and paper surveys.

The search for methods that bridge this identified time lag have pointed to digital approaches. In recent years health services have been increasingly digitised with a concomitant expectation that patients will engage with digital systems [2, 5, 6]. Moving towards digital patient feedback is in keeping with this trend, and it offers the advantage of speed (timeliness) and reduced costs. Further, there is good evidence that digital systems can be empowering for patients with some long-term conditions to provide essential self-management information and services [6–8].

At the same time, digital capture poses a number of challenges, such as clarity about its nature and purpose [9] and how health organisations can best use this type of data [10]. Critical literature has also emerged to consider the potential for digital inequalities and barriers to participation, as well as how wishes and preferences for engaging with digital technologies and sharing health data may vary according to social and organisational contexts [11, 12]. A recent paper examining the adoption of new communication technologies by older people provides insights that can be applied more generally: the main determinants for adoption relate to attitudinal, functional and physical factors [13]. Furthermore, contextual and socio-technical dimensions are important to consider alongside human agency and people’s lived experience of technology [9, 12]. While these issues relate to a wide range of digital modalities, they are pertinent to patients’ adoption of digital feedback tools and we will include them in our conceptual analytical framework.

A further issue is the perceived ‘usefulness’ of digital feedback. Coulter et al. [14] identify the lack of impact that patient feedback appears to have on change in the NHS, and others have pointed to the need for researching the actions and interactions necessarily entailed to create and use varied forms of feedback (quantitative and qualitative) as a means of understanding how data travels and becomes transformed in relation to quality improvement agendas within complex healthcare environments [15, 16]. This link between the collection and analysis of feedback data and any change in service provision appears to be important in terms of both staff and patients’ engagement with providing feedback, whether it is digital or otherwise [17].

The introduction of the digital collection of patient experience data can be disruptive in multiple ways: to clinician-patient relations, to performance management and to wider governance systems in healthcare. Consequently, there is a need to attend to the way in which digital approaches are introduced. The focus of this paper is on the implementation of a particular digital feedback intervention that was co-designed with health professionals and patients (the co-design approach is reported elsewhere). We will describe the nature and scope of the intervention, the contexts within which it has operated, how the different participants made sense of the intervention and engaged with it (or not). Whilst our study focused on the implementation of a specific digital based system for collecting and using patient feedback it serves as an exemplar with wider resonance for systems requiring engagement of patients and staff with digital tools in clinical setting. The evaluation of the overall process of implementation used Normalisation Process Theory (NPT) and here we discuss the extent of adoption and routinisation that has taken place within the different contexts, and the reasons why differences emerged.

Digital interventions are viewed as complex because they include several interacting components, including changes in individual, group, systemic and organisational behaviours [18]. Many conceptual models have been developed over the last few decades to understand and evaluate complex interventions [19–22], and digital health has become a fruitful area of study. Authors have highlighted the need to focus on multiple complex components when evaluating digital health interventions including changes in therapeutic interactions and behaviour, and the degree to which the intended population is engaged [7]. Relevant research has drawn attention to key strategies to support implementation for digital interventions witin routine healthcare including developing links with key leaders and the use of educational materials, as well as feedback and reminders [23]. Emerging literature evaluating digital innovations for enhancing audit and feedback within ‘learning health systems’ is particularly relevant to this study on digital patient feedback. Such literature has also drawn attention to major organisational barriers that mitigate successful implementation and outcomes from innovation [24]. Similarly, literature from Information Systems and Healthcare Information Management fields have also pointed to the importance of organisational environments and system readiness (especially interoperability, [25]) for new information systems. Such literature has demonstrated that small organsations face greater barriers, and communication with front line leaders have a positive influence on diffusion and adoption [26, 27]. However, such studies have also highlighted the need to consider the tacit nature by which knowledge is accumulated by medical professionals and the need to ensure new knowledge systems are able to be integrated with such unstructured knowledge for successful adoption [28]. Studies have also provided some insights into the effects of e-health interventions on roles and responsibilities of participants (professionals and patients), how these interventions affect clinical activities and interactions, and the allocation and performance of clinical work [29]. However, Mair and colleagues [30] completed a systematic review of the implementation of e-health using the Normalisation Process Theory [31, 32] and argued that the emphasis in most studies has been on the workability of systems but relatively little attention has been paid to the micro-interactions relevant to clinical work and practice. It is precisely this gap that the current paper attempts to address: the different ways in which digital interventions are interpreted by the parties involved and how these perceptions shape the way in which professionals and patients engage with the new technology. The multiplicity of actions and interactions arising from this sense-making represent the work involved necessary to align the digital approach to the routines of the various clinical contexts. In order to surface the intricate processes involved, and taking account of the differences between acute, mental health and primary care settings, a robust conceptual framework needs to anchor the analysis and in this project the Normalisation Process Theory has been selected.

NPT is a middle range theory that allows for a multifaceted analysis to understand the actions and interactions influencing implementation and how new interventions and practices come to be normalised in health care contexts; or serves as a means of explaining why technologies fail to be routinely adopted when implemented in organisational contexts [22]. NPT is built around four key constructs: first, *coherence* refers to the meaning and understanding of a new technology and its associated practices; second, *cognitive participation* refers to the relational work needed to sustain a community of practice for a new intervention; third, *collective action* refers to the operational work to enact new practices and fourth, *reflexive monitoring* refers to the work done to monitor and appraise new practices.

Two systematic reviews [33, 34] argue that the NPT constructs provide a conceptual framework to highlight important issues relating to routinisation which is a key element additional to previous theories in order to understand not only implementation but also how complex interventions become embedded in everyday practice. It is particularly appropriate for examining individual and collective behaviour in the processes of change, and allows the dynamics of human agency to be connected to context. The reviews conclude that the NPT constructs are stable and consistent, and that they are flexible and understandable across contexts. Futhermore, NPT has explanatory power and is particularly useful because it opens up the process of ‘work’ that is required for adoption and integration of new interventions. Given that our study focuses on examining the strengths and weaknesses of a new digital intervention, its implementation and routinisation, NPT provides an appropriate conceptual framework. We will illustrate below (Table 2) how the NPT constructs have been operationalised in our study.

## Methods

This paper reports on the implementation and evaluation of a digital patient feedback system within 4 health service organisations (Acute Trust, Mental Health Trust, and two General Practices). This component was part of a larger mixed methods study entitled ‘Developing and Enhancing the Usefulness of Patient Experience and Narrative Data [35]. The DEPEND study aimed to understand how to improve the credibility, usefulness and relevance of patient experience data in services for people with long-term conditions using digital data capture and improved analysis of narrative data.

The DEPEND study comprised multiple work streams including qualitative exploration of perspectives of patients and carers and staff on the collection and use of patient feedback for service improvement. A text mining component^1^ aimed to develop routine, semi-automated analysis of free text feedback comments. These aspects then informed co-design of new tools (digital and non-digital) tools to support the capture, analysis and use of patient feedback. The new tools comprised: a survey to complete digitally via tablet device (a self-standing kiosk or a tablet sat on a reception desk) in waiting areas or pen and paper/online version; guidance and information for patients, carers and staff; reporting templates; a process for eliciting and recording verbal feedback in community mental health services. The final phase of the study which is the focus of this paper entailed implementation of the aforementioned tools and a process evaluation of the new tools using NPT.

The initial research questions pertaining to the implementation of the toolkit were:

How was the toolkit introduced in the four settings? How did the different staff groups respond to the toolkit and were any changes made to the toolkit and its operation? How did patients and their social network respond to the toolkit? Did staff and patients compare the new approach to existing methods of feedback?

### Participants and data collection

The four research sites which were selected to ensure diverse organisational contexts and variations in terms of methods of collecting patient experience feedback. Within the acute trust we focused specifically on a rheumatology outpatient clinic (site A). Within the mental health trust we focused on an outpatient department (OPT, Site B) as well as a Community Mental Health Team (CMHT, Site B). The two participating general practices (Sites C1 & C2) were in the same localities (C1 in the same area as site A; C2 in the same area as site B).

All sites collected feedback via the Friends and Family test but most relied on pen and paper to do this and there were low levels of participation. There was limited digital data collection via text messages in two of the sites and none were routinely collecting feedback digitally within waiting areas on site.

Participants in the implementation and evaluation component reported here included staff, patients and carers. Following introduction of the new tools qualitative data were generated in a number of ways: large focus groups that through interactions allowed a multi-dimensional perspective to emerge; individual interviews that provided in-depth personal narratives as well as observing practices and patients/carers in the use of the kiosk. Further details of participants and methods are reported elsewhere [36] but to summarise, there were 51 staff participants, 24 patients and 8 carers in total from across all sites. The samples reflect maximum variation [37] by including a balance of patient and carer participants in terms of gender, age, ethnicity, lived experience as far as possible. For staff sampling, we ensured diversity in terms of roles and experience. The study design allowed for in-depth investigation and enabled triangulation of the data and emerging key themes.

Focused discussions with members of our Patient and Public Involvement (PPI) Advisory group were undertaken to incorporate their insights on the unfolding qualitative data, co-design of the new tools, as well as the evaluation of the study. This group included people with a range of relevant and diverse health experiences. We also worked with two pre-existing Patient and Participation Groups (PPGs) that serve as involvement and advisory groups within the participating primary care sites (C1 and C2).

### Data analysis

Transcripts from interviews and focus groups, and field notes from observations were collated and analysed thematically using a grounded theory approach [38], whose fundamental tenet is that the emergent theory is grounded in inductive data analysis. An important difference with the original conception is expressed by Charmaz: “Unlike their [Glaser and Strauss] position, I assume that neither data nor theories are discovered. Rather we are part of the world we study and the data we collect. We *construct* our grounded theories through our past and present involvement and interactions with people, perspectives and research practices” ([39], p.10). In our case this means that while our thinking is shaped by theories of complex interventions, we have consciously adopted an open approach to the analytical process. The modification in our study entails that on the one hand accepted conventions are used such as theoretical sampling, constant comparisons and thematic analysis, but on the other hand the findings from the inductive analysis are compared to the NPT constructs in order to allow for a deeper understanding of the processes of adoption and routinisation. The coding and analysis have been aided by using NVivo11 qualitative analysis software.

The next step was to translate the NPT constructs to make them relevant to our study through team discussion of each construct and brainstorming in order to identify specific questions pertaining to the digital intervention and the different contexts [40]. These specific questions were ordered in tabular form (see Table 1) to facilitate mapping the results of the inductive analysis and emerging themes against the NPT constructs so that a coherent conceptual analysis could be developed. Finally, the volume of patient experience data before and after the introduction of the new tools were quantitatively analysed as part of the overall process evaluation.

**Table 1:** Normalization process theory coding framework used for qualitative analysis of digital patient feedback implementation

Coherence	Cognitive participation	Collective action	Reflexive monitoring
**Differentiation:** Is there a clear understanding of how digital feedback differs from existing practice?	**Enrolment:** Do individuals “buy into” the idea of digital feedback?	**Skill set workability:** How does the innovation affect roles and responsibilities or training needs?	**Reconfiguration:** Do individuals try to alter the digital feedback system?
**Communal specification:** Do individuals have a shared understanding of the aims, objectives and expected benefits of digital feedback?	**Activation:** Can individuals sustain their involvement?	**Contextual Integration:** Is there organizational support?	**Communal appraisal:** How do groups judge the value of digital feedback?
**Individual specification:** Do individuals have a clear understanding of their specific tasks and responsibilities in the implementation of digital feedback?	**Initiation:** Are key individuals willing to drive the implementation?	**Interactional workability:** Does digital feedback make people’s work easier?	**Individual appraisal;** How do individuals appraise the effects on them and their work environment?
**Internalization:** Do individuals understand the value, benefits and importance of digital feedback?	**Legitimation:** Do individuals believe it is right for them to be involved?	**Relational integration:** Do individuals have confidence in the new system?	**Systematization:** How are benefits or problems identified or measured?

## Results

The initial qualitative research in the first phase of the DEPEND study and the co-design approach meant that an understanding of sense-making amongst patients, carers, members of the PPI group and staff underpinned the development of the toolkit that was tested out in the fourth phase of the DEPEND study. The close involvement of participants from the outset and during the lifetime of the project meant that this sense-making could be a continuous process, and was not confined to the start period.

### Coherence: making sense of new digital feedback tools

#### Health care staff

Staff members in three of the four sites (A, B, C2) were generally enthusiastic about the introduction of new tools for collecting feedback digitally on site, including the kiosk. They felt that the digital collection of feedback might improve the volume and efficiency of routine patient experience data collection as reliance on pen and paper surveys had failed to provide meaningful information:

‘What we currently collect is box ticking and none of us find it useful’ (Site C2, FG1, ID335, Lead GP)

Moreover, response rates had tended to be low and feedback delayed, so reacting to suggestions or complaints became slowed down, and in turn, could cause a feeling among patients that providing feedback did not lead to any organisational action.

The degree of optimism in how well digital feedback might actually work differed between the sites, and within the mental health context the complexity of clinical care was highlighted and having a simplistic mechanism to enable routine feedback was seen as appropriate:

‘[ …] quite often I imagine in the day to day contacts with the care coordinators the time is so absorbed with their illness or what's been going on in their lives that they don't have that time to even ask (about the service received), or it might not be appropriate to ask that question. Whereas this is a choice, isn't it? I can choose now to say how I feel about our service in a very simplistic way’ (Site B OPT, FG, ID 243, Senior administrator).

The distinction between traditional feedback methods and the digital approach was taken further by another manager in terms of the benefits to service itself:

‘… what always really, really frustrated me was that lack of ability that we had to then funnel that back down into services. So [team lead name] actually was usually one of the ones that would always come to me and say come on, now, we've sent you all of this data, all of this information about our team, our service users, what is that telling us? … at one point I was doing all of these bespoke reports…it became very apparent very quickly that I just could not do that… And I think what (The DEPEND project) always I suppose promised, or had the potential, was the ability to do that bit that was missing.’ (Site B CMHT, interview, ID 201, Senior manager).

The comparison made between the old system and the new digital method allowed this manager to see the potential benefit of all the data to be collated within one system and thus offering the possibility of linking patient experience with service improvement in a more targeted way. Furthermore, the analytical work was perceived to be reduced by the potential for enabling a semi-automatic system.

In the primary care setting, the two participating practices had different perspectives on the value of digital feedback, with one (Site C2) being very positive and highly engaged throughout the evaluation phase and project, which was most likely related to the close involvement of a senior partner and the practice manager with the project, and an active PPG attached to the practice. Conversely, in the other practice (Site C1), engagement was much more limited and the perceived potential limitations of digital data capture were emphasised. Of note, a lead GP during his interview referred to his own experience of giving patient experience feedback when he said:

‘Well to be honest with you, when I go to my GP, I don't go that often but I do go. I can't fill it out [the FFT] until I’ve been in because I don't know what my experience is do I? And when I come out I just want to go… ’ (Site C1, interview, ID141, GP).

This comment reflects that patients weigh up spending time on filling in digital feedback with getting on with their lives, and value the latter higher. This was also reflected in observation notes when many patients commented that they did not have time to give feedback, but also would probably not give feedback via text or internet at a subsequent point. As such, this may not be specific to the digital nature of the feedback sought, but more generally to engaging with the process. However, it indicates that offering a digital option may not make much difference to this type of patient.

In the DEPEND study, the digital data collection took place via a kiosk placed within clinic waiting areas. Primary care staff made comparisons between pen and paper methods and the kiosk with the latter being seen as potentially attractive to patients and carers:

‘… So as opposed to pen and paper or something like that. I think it's quicker, easier to do it on there [via the kiosk] to give feedback’ (Site C1, FG, ID134, Practice Manager 2).

One of the key reasons that digital approaches made sense to staff was their feeling that pen and paper methods failed because they found the papers just littered around the waiting room and the feedback boxes remained empty. While it was thought that giving feedback on a tablet might be easier this did not necessarily impact on actual participation as we will show in the next section.

#### Patients and carers

NHS patients and carers are now commonly asked to provide feedback on services received, and understand that this is increasingly done digitally. Many people mentioned that they recognised that for organisations this format is preferable, but several older patients and carers expressed reservations such as a general reluctance to engage with digital technology:

‘ I’ve got a computer at home, but the thing is, I don’t like the digital age ‘cause I’m only learning a bit more on the computer. I had to go for a course on it actually, a beginner course, you see? ‘Cause when I used to work, we used to work on stock systems you see, that was years ago, in the 90s, but then I haven’t been able to work since about 2001 you see? Cause of this illness you know? But the thing is, I’m not so used to digital stuff, like whizz kids are, like these youngsters today. I’m going to learn a bit more…’ (Site B OPT, FG, ID 247, patient).

It was recognised that marked variations in uses and preferences regarding digital technologies existed. For those routinely engaged with this world giving patient feedback digitally made sense, but it could be very difficult for others. One staff member talked about their own personal experience:

‘I think a lot of people these days are familiar with using that kind of technology and so… and the majority of people are comfortable and like to do it in that way. I know that if it was me I would rather, you know, work on something like that than paper and writing things down… on the flipside of it, you know… it scares some people… I’m just sort of going off really sort of personal experience of people that I know in my…home life who are experiencing some difficulties, you know, with mental health or age related’ (Site B OPT, interview, ID 242, Administrator).

The suggestion of offering alternatives alongside digital feedback was echoed across the patients in this study. Suggestions were made to keep the option for giving written feedback:

‘…you’ve got to think of the other person that prefers maybe just getting a bit of paper and saying, ‘oh, I was happy today’, fold it and throw it in a box’ (Site A, interview, ID223, patient).

Or to allow patients and carers to provide verbal feedback:

‘…because sometimes people can talk about it more than write’ (Site A, interview, ID223, patient).

It can be argued that while digital feedback is seen by some patients as the logical way forward a number of factors inhibit the way digitalisation makes sense within their everyday context: individual preference for written or spoken feedback, and reluctance or lack of confidence to use digital methods. This variability needs to be better understood in order to develop approaches that are coherent with people’s life world.

### Cognitive participation: driving digital feedback forward

#### Health care staff

Individual and collective investment in the new digital feedback approach varied across the four sites. The level of ‘buy in’ at the two GP practices differed because in site C2 a lead partner and the practice manager believed that the kiosk was a positive way forward. In this site the kiosk was actively advertised and the PPG became involved from the outset of the study as part of the patient and public involvement component of DEPEND (this crucial insight and involvement to the co-design of the new tools is reported elsewhere). As a result, new ways of advertising the study were discussed and agreed with the practice team and PPG in order to encourage patients and carers to feedback using the new tools. This specifically worked in practice as this PPG saw their active involvement as appropriate to their role and function. Observation notes made during a PPG meeting within site C2 attested to their proactive collaboration with clear allocation of tasks to move implementation forward. The agreed actions below were designed to help sustaining the intervention over time:

- ◦ Practice Manager (PM) will upload project information on to the Practice Facebook page as a new method of advertising the toolkit to get feedback on specific clinics.
- ◦ Advertisement and the next steps (including testing out bespoke questions to gather useful data that will help with staff revalidation) will be discussed in next week’s staff meeting.
- ◦ The PM and project researcher will pick this up at the next PPG meeting.
- ◦ The PM to place a project advert in both the practice and PPG newsletter.
  (Site C2: Observation note)

In site C1 some members of staff considered the kiosk a good alternative to current feedback methods because the collection of patient perspectives was important and pen and paper methods had failed previously. However, stimulating the use of the kiosk was not seen as part of their job:

‘No, we haven't got the time for that. So…and I'm going back a few years now because a few years ago patient surveys were quite high on everybody’s agenda. You know, we had to do it for the NHS, we didn’t have a choice. So we had to do an annual, you know, survey but they stopped all that. So if… well to us, because we're so busy anyway, if we don’t have to do it and we're not getting paid to do it, sorry, but we're not going to do it. I'm only being honest’ (Site C1, FG1, ID 139, Practice Manager 1).

In contrast, staff in the other sites mentioned the increasing pressure they felt regarding the obligation to collect patient feedback, and in site C2 this was referred to as a contractual obligation. They also expressed preference for collecting de-anonymised data for staff revalidation which was beyond the project’s remit.

The issue of time pressures came up regularly and neither the clinicians nor the administrative staff felt that they had the capacity to explain to patients that this new kiosk was available. Clinicians said that during the consultation priority had to be given to the many essential tasks which needed to be completed. Receptionists were considered overburdened and asking them to add alerting patients to the kiosk would ‘would probably tip them over the [edge]’ (Site C1, FG ID 134, Practice Manager 2). In the same site, the research team found that the kiosk had crashed and no data had been collected for some time. This was a technical glitch that could be resolved by re-booting and the team asked whether someone could routinely check (for example, each morning) that the machine worked. The receptionists said they would do it, but in practice this never happened.

In Site A (Acute Trust) a reasonable level of coherence emerged and recognition that testing out digital tools to support data capture made sense at the outset. However, during the testing period a number of staff indicated a degree of detachment from the tools being tested, including the kiosk. They expressed concern that they did not think it appropriate for them to be seen as ‘promoting’ or driving the sustained use of the kiosk. Furthermore, some clinicians at this site thought that advertising the use of the new kiosk might introduce a ‘bias’ in the sentiment of the feedback because patients might think they are being asked to give positive feedback about their individual clinical practice.

In Site B, the Mental Health Trust, a number of comments made by the staff in the community mental health team indicated barriers to sustaining a community of practice to drive the new process for recording verbal feedback forward. One of the main issues that emerged was related to the complex and individualised situation of patients using mental health services. Judging whether to use an iPad to collect feedback on home visits was deemed difficult:

‘The ones that want to sort of engage because I know some, you know, when I'm there they get really distressed and that's the last thing they want to do. I've got one lady in particular I wouldn’t use it [iPad] with, and it depends on where they are as well at the moment, I suppose, within their mental health, and she's very sensitive to anything…’ (Site B CMHT, FG1, ID 215, Care coordinator).

While this care coordinator thought digital feedback was a positive development actually using it in practice was contingent upon assessing the state of mind of the patient, and thus participation could not be generic but varied from individual to individual. The mental health service users reiterated this perspective in the focus group discussion.

Staff also talked about how during the testing period the new system for recording feedback seemed to slip from their agenda. In the busy outpatient department it was easy to overlook this new approach amongst established routines, and with staff turnover in a large team information did not readily get passed on. There was a lack of relational work to stay activated in collectively defining and sustaining the practice:

‘I've got two clients that I see. What we have agreed, and is what we agreed with staff, is that we should be asking the question about how a person has felt the encounter to be and to record it in ‘plan’ [referring to specific field in the electronic record]. What we've also acknowledged is that individuals will ask that at whatever frequency they feel is appropriate, but what we encourage them to do is to put in plan, question not asked, if they don’t…. Now I've been asking staff in supervision and they’ve forgotten and, candidly, for my own part with my two patients, I have as well’ (Site B CMHT, interview, ID 341, Manager).

Despite many of the staff considering that supporting digital patient feedback was part of their role and that they would act accordingly, the everyday pressures of clinical practice meant that they often did not remind patients and thus sustaining the new practice was uneven.

#### Patients and carers

Patients and carers were generally positive about the introduction of digital methods, but mentioned that it could never be a ‘one size fits all’ method. A number of barriers to participation were mentioned, such as literacy, current health status, specific disabilities or individual preferences for giving feedback, and thus alternative approaches should co-exist:

‘I think the general principles should be that if you're going to go down the digital route absolutely do it, it is the future, it works for the majority, but don’t ignore the need to do some of those other additional means that will need to be more targeted’ (Site A, interview, ID 123, patient).

Concerns about confidentiality were often voiced, especially by patients with long-term conditions for whom good relationships with health care professionals were important. The advantage of digital means was expressed as follows:

‘Probably I think with it being done on a tablet or on a phone if you do…if you're picking the answers on there, there's no way that anybody could know that it was you because you're in the comfort of your own home or they couldn’t know it was you that wrote it and if you didn’t have to log in with your details, or something, probably be more honest to answer questions’ (Site A, interview, ID 119, patient).

Although most patients talked positively about the kiosk, actual use of it did not reflect this attitude. Observations in the clinics showed limited engagement and various barriers were described at interview, such as:

‘Okay, I recently had a rheumatology appointment and I couldn’t actually see the machine. I had to ask somebody where it was and where it was positioned, I think was a bit awkward, it’s not really visible. I then, went over to… after I’d asked somebody where it was, I then went over to try and record my feelings and it wasn’t working, it said it had logged out or there was an error or something like that’ (Site A, interview, ID107, patient).

The placement of the kiosk was discussed at length with the various sites as it became clear that it had to be accessible and inviting, but at the same time offering sufficient privacy (see also Ong & Sanders [36] where issues regarding the spatial issues and micro-interactions with the technology are analysed and discussed in detail). Monitoring that the iPad actually worked and maintained was equally important, but proved to be a bone of contention with staff, and thus it impacted on patients’ participation:

‘No, as far as I’m concerned it’s a research project, I don't know what our responsibilities are, but 1) I wouldn't touch it with a bargepole because I wouldn't know what I’m doing and 2) it’s not within the remit of our cleaning staff. You’re talking about cleaning, to clean it. So nobody’s going to clean it’ (Site C1, FG, ID 141, Lead GP).

The differences between the sites were clearly related to whether or not key individuals drove the new feedback approach forward. As previously noted, the involvement of the PPG in one of the primary care sites proved to be a crucially positive factor during the implementation of the digital patient feedback system. A core PPG group were regularly present in the practice and two members actively supported individuals to use the kiosk. In contrast, not many staff considered it to be their role to support patients to use the kiosk or to ensure that it actually worked. Site A was a case in point where an attempt was made to engineer a system for getting nursing staff to direct patients to the kiosk because it was placed off to the side. Following some discussion about how best to sign post the kiosk the nurses in the treatment room where patients get their blood tests done said they would hand information slips to patients and then direct them where to find the machine. Again, this was not borne out in practice. It was clear that sustaining interest in the digital system fluctuated and as a result of the various influencing factors staff participation was variable between sites.

### Collective action

#### Health care staff

The organisational context to continue supporting digital feedback is key at this stage and consistent commitment of leaders within organisations can ensure that participants maintain their belief and engagement in the new intervention.

Across all organisations in the DEPEND study the issue of workload emerged as a main determinant, and mirrored some of the concerns mentioned in relation to cognitive participation. These concerns appeared to be more pressing in relation to collective action because staff recognised that collecting feedback should become part of routine practice, and they felt this was difficult:

‘Because of staff absence, people are having to pick up other people’s work to some extent or another, on top of their own work, and they’ve already got really high caseloads’(Site B CMHT, interview, ID 216, carer support worker).

The daily reality for health care staff was the lack of ‘slack’ in the system and taking on extra tasks was seen as problematic. In the mental health site, staff had experienced major changes in the structure and leadership of the organisation. Particularly within the community mental health team, many problems existed with long-term sickness and a high staff turnover, such that the team seemed unable to collectively build a shared accountability for the new process for recording verbal feedback.

In addition, primary care staff mentioned that many patients did not go to the kiosk on their own initiative:

‘No, I mean, I think the kiosk is fine, I think it's just getting the patients to use it. You know, unless you actually stand it in the middle of the door where they're going to trip over it, I don't think they're using it’ (Site C1, FG, ID 139, Practice manager 1).

Clinical staff were unsure whether promoting the use of the kiosk was part of their role:

‘…by the time you've done your consultation and gone through all the various bits, then we might remember. But it [requesting feedback via the kiosk] would be very hit and miss if I'm honest, because you're usually quite pleased if you've managed to make all the boxes go away, as well as do a consultation. And I would imagine, certainly at my age, I'd forget’ (Site C1, FG, ID 141, GP).

While this GP said that he had intended to mention the kiosk other tasks were prioritised and requesting feedback was forgotten because it was not considered as core to the consultation.

In the acute site, timing of feedback was mentioned as a barrier to asking people to use the kiosk and some clinicians reasoned that it was ‘unfair’ to ask people to feedback directly after a consultation:

‘I don’t think many doctors are mentioning their patients to provide feedback… I had a brief chat with Dr [name], she thinks it’s better if I tell the patients to give feedback instead of doctors telling them, otherwise it might influence the patients’ feedback’ (Site A: observational note).

As mentioned earlier, consultants in this site felt it was an ethical concern for them to ask patients to give feedback following their outpatient consultation. Clinicians also admitted to not having seen the flyers advertising the new feedback tools despite the research team and clinical lead on the project giving these out during observational sessions and at team meetings:

‘These little flyers here, I’m ashamed to say I’ve not seen these before. Where were they?’ (Site A, FG, ID 321, consultant).

Taking these comments together a picture emerges of at least some staff intending to encourage patients to use the feedback system, but both at the individual and collective level systematic action was missing. The main reason appeared to be that staff considered alerting patients as an optional ‘add-on’ to their everyday work and the structural conditions with high workloads often mitigated against consistent behaviours.

One way of addressing this problem was highlighted by focusing on the selection of the ‘right’ staff to maintain the momentum:

‘So, on a ward, it might be a staff nurse, in a podiatry setting it might be the podiatrists, on the ground, or the reception staff. So wherever you think that it would be easiest to collect that back, that’s where you’d pitch it, you wouldn’t think of the role of that person as being massively more- anyone can feed back into the improvement work…’ (Site A, interview, ID 105, Senior manager).

The connection between specific service feedback and the ‘ownership’ by staff within that service was suggested to be a positive motivating factor for coalface staff to remain involved.

Another factor that played an important role in the uneven uptake was technical because staff in all sites mentioned malfunctioning of the kiosk, including freezing of the screen, unreliable WiFi connections, and the iPad not saving inputs. It was mentioned that the response time of the company supporting the technology was inadequate, thus reducing engagement with the kiosk and staff losing momentum.

In the community mental health site, specific structural and staffing factors mentioned earlier were highlighted relating to the content of their work. This affected the team’s ability to collectively build a shared accountability for the new process for eliciting and recording verbal feedback:

‘I think because we don’t work to clear cut pathways and because staff are given a selection of training that's considered to be appropriate for the role but is never keynoted to a particular intervention as such and because people's caseloads are so diverse then I think there is little meaningful link between training and direct action’ (Site B CMHT, interview, ID 341, Senior manager).

It was clear that community mental health team members faced multiple barriers to operationalising the new process, not only in terms of allocating the work within the team, but also in building confidence in the new practice. In addition, they faced difficulties in adapting the existing care record technology:

‘Well the, I mean, the area we'd identified was, needing to put the narrative response in… the box… And I think the problem… was about a question… And at the time we sort of did it as an open exercise, to practice… then it became an issue of, well which type of question… perhaps the ambiguity as well of how work is conducted can make it difficult because if there is an ambiguity of purpose then it's harder to ask a question, and, equally, what kind of the answer are you going to get if the person on the other side equally shares that ambiguity?’ (Site B CMHT, interview ID 341, Senior manager).

Thus, the combination of structural and technical changes limited the adaptability of the system and the staff working within.

When looking at the factors that facilitated collective action the DEPEND team in partnership with the project PPI advisory group developed a number of interventions. They created clear and easy to understand summary reports for each site that were presented at regular intervals. This helped staff to digest feedback and formulate any actions resulting from their discussions. For example, the signposting to the kiosk was changed in one of the primary care sites and instructions were made clearer resulting in more patients using the kiosk. The link between feedback and service change could thus be demonstrated and was experienced as positive.

In site C2, the consistent involvement of the PPG was instrumental in maintaining confidence in the new system. Through their direct engagement with patients and carers they could pick up any problems and issues and with the practice manager and DEPEND team find solutions. Through dynamic adaptation, the kiosk remained relevant to both patients and the practice. In the other sites, these processes were more inconsistent and more attention was paid to the mechanisms that prevented sustaining collective action.

#### Patients and carers

Patients’ continuous engagement with digital feedback depended on structural factors such as the visible and inviting positioning of the kiosk. Technical hitches could discourage people such as in the following example from the acute trust:

‘One patient was willing to give feedback after getting consultation from the doctor while she was still waiting for blood test to be done. When she was typing on the Kiosk, the nurse called her for blood collection. She then left the kiosk [and] …the page that she was typing disappeared when she returned from the blood room. She had to type everything again’ (Site A: observational note).

Instead of making it easier for patients to give feedback the technical problems made it more difficult and thus threatened continued use of the kiosk. This could be mitigated if a staff member was trained in resolving these issues, but none of the sites had identified such a person and thus dependence on an outside agency to respond hampered quick resolutions.

In site B specific issues relating to people’s mental health were highlighted that could influence engagement with the kiosk. First, mention was made that when individuals were unwell they would not want to provide feedback, or may have negative feelings about the technology:

‘…sometimes they can still be quite unwell […] some of our clients that come, there is a lot of, I guess, paranoia about the sort of technology in that […] devices that are digital, they think that somebody is trying, you know, that there are people trying to communicate with them through these sort of devices’ (Site B OPT, interview, ID 242, Administrator)

Second, individuals might be stressed or anxious which affected how they perceived the service and they could be confused about the reasons for attending. Asking them to provide digital feedback would be problematic:

’ …they have got no idea which service they are here to see, so as soon as they get confused they back up… I think it [kiosk] is overcomplicating things’ (Site B, FG, ID 254, CPN).

The need to understand the individual patient and their state of health was deemed to be crucial to gauging their willingness and ability to actively engage with the digital technology. The support and guidance of a trusted professional or volunteer (Site B, OPT) could help to overcome personal barriers. In summary, patients’ confidence in the new system was shaped by a range of factors, including structural and technical workability alongside individual conditions and health status.

### Reflexive monitoring

#### Health care staff

This NPT construct is concerned with participants’ perspectives on the effects of the digital feedback tools and whether they consider it worthwhile, individually and collectively, to continue its use. Furthermore, an assessment is made as to the changes that are made to working practices in order to embed digital feedback as routine.

While staff appeared generally positive about the potential of digital feedback, no significant changes to service delivery were observed during the study period. This was unsurprising due to the relatively short evaluation period, but also influenced by the uneven adoption across the sites. Yet, some medium-term changes could be identified and, for example, in the acute trust the potential of the new approach was seen to fit with an overall shift in the use of patient feedback:

‘…. and you’re sometimes using it as a little bit of a balancing measure in some instances, so you’d want to make sure that the patients were central in something you’re thinking around. You might be trying to make something more efficient, or you might be trying to increase the number of patients that you could see through an outpatient clinic, or making something more efficient might contradict with what the patient wants, so you just try and balance that out, and consider them, each patient, to make it as patient centred as possible’ (Site A, interview, ID 105, Senior manager).

The monthly DEPEND reports summarising feedback prepared via the kiosk were considered to be useful by staff and were discussed in monthly feedback at team meetings -

‘I think this will be very useful. I mean we've just had a governance meeting so presenting this every month would be very useful’ (Site A, FG, ID 131, Specialist Nurse).

Having the feedback analysed and presented in this simple format stimulated discussions about potential changes to their clinical practice that was often missed from the generic feedback previously collected. The team expressed a preference for tailored questions over the generic FFT question in order to elicit specific and detailed free text comments that could provide pointers for service improvement -.

‘… The question can be asked differently, ask them specific questions, like [about] access… Only then we may be able to use the report to improve this department’ (Site A: feedback meeting observational note).

The above reflections pointed towards the possibility that digital feedback could become embedded within the organisation and adopted as routine practice. In the primary care sites the potential of digital feedback was viewed positively. First, a suggestion was to use clinician specific feedback for revalidation purposes or clinical training.Second, the physical presence of the kiosk and structural change in the PPG peer support role combined could prove important factors that facilitate the adoption of digital feedback as routine:

‘So I think the kiosk has been positively viewed… I can't think of any negative comments about the kiosk. There may have been, but I'm not aware of them. It's generally been positively viewed. It seems to have been used by quite a number of people. I think it's still got potential to have… to be used more for different and innovative things, but as a first trial it seems to have worked very well and people seem to be engaging with it’ (Site C2, interview, ID 335, Lead GP).

A further suggestion to enhance the visibility of the kiosk was, for example, placing it in the pharmacy where people could us the kiosk whilst waiting for their prescription. Given the trend to integrate primary care services this sort of development could aid more routine uptake of the kiosk.

The least strong indications that a new approach to eliciting and recording digital feedback could be embedded in everyday practice were identified in the community mental health team even though the digital system was adopted in out-patients. The main factor was the major organisational change taking place during the DEPEND project that had structural and personnel implications. On the one hand, the potential of the new approach was clearly recognised:

‘I think as an organisation it’s been a hugely helpful experience really. I think what it’s obviously told us is we really want to generate the quantity and create something that’s smart and it’s easy then to filter back down and attribute to individual wards and all the rest of it’ (Site B CMHT, interview, ID 201, Senior Manager).

Yet, countervailing forces were perceived to be major inhibitors to adoption and routinisation of the new system:

‘I think generally all of that change as we went through as an organisation, if you think about that in the context of [Lead’s name] team, I think from a timing point of view it was probably really unhelpful for the research. Because I think what was happening, certainly in my case there was change, there was change in circumstances, there was new priorities. There were new things that we needed to focus on as an organisation. And I think sadly what that meant was there were a lot of things that we just couldn’t do anymore, or that became really difficult to do because there were new things, other things that we had to worry about’ (Site B CMHT, interview, ID 201, Senior Manager).

This view was echoed all the way through to the coalface with support workers saying that the introduction of new care record systems took up a lot of time and while they wished to embed the new feedback mechanisms, they did not have the capacity to do so. This was evident for both the new system for recording verbal feedback within the community mental health team, and the limited use of the digital iPad in the reception area of the outpatients clinic for the mental health Trust (site B). Several reasons for low participation rates were described by Outpatients staff including: the location of the kiosk sat on reception raising privacy concerns; the different clinics on offer which tended to involve longer appointments, and less people to approach to ask to use the kiosk; as well as a general feeling of disengagement to the feedback system, mainly due to literacy concerns, with hardly any feedback collected via the different methods.

‘So we kept an eye on it [iPad screen] but alongside the cards [FFT postcards] going into the box. But as time’s gone on I’ve asked the reception girls who I line manage and they said, ‘oh, more and more people are asking about it’ and the clinic clerk, [name], is promoting it as such, but without over-promoting it. Because I think what’s difficult in our reception area is it’s so small and sometimes people don’t want to be put on the spot in front of other people’ (Site B OPT, interview, ID 243, Senior administrator).

#### Patients and carers

In the primary care site where the PPG played an active part in the DEPEND study their role was re-evaluated and it was agreed that they would remain involved in supporting patients and carers in providing feedback beyond the study period. This was an important development as a proportion of patients and carers felt they needed help with the new technology, even though the general perception was that digital methods were acceptable and helpful.

## Discussion

The DEPEND study aimed to implement a new digital patient feedback system in four different health care sites. The new system was considered a complex intervention and therefore using the NPT as the conceptual framework was appropriate. The four constructs allowed for a detailed analysis of the actions and interactions taking place within the different sites in order to embed the digital method.

With regard to sense-making staff and patients understood that the kiosk constituted a fundamentally different way of collecting patient feedback. Yet, no communal shared perspective emerged as individuals defined the purpose and benefits in a variety of ways and this reflects the spectrum of views reported elsewhere that have questioned the ‘meaning’ and purpose of patient feedback and other forms of data used within quality improvement initiatives [16, 41]. Consequently, a range of interpretations of the tasks associated with introducing the kiosk ensued and taking responsibility for implementation varied between the sites.

The sites demonstrated different degrees of cognitive participation, and their commitment to the digital approach varied from, on the one hand site C2 having a lead GP and practice manager to champion it, and on the other hand in site C1 no particular individual was championing the case. Clinical and reception staff were not agreed whether it was their role to ask patients to give feedback, and even if they agreed to stimulate participation it rarely happened in practice. These issues draw attention to the importance of leadership, information and support and especially for innovations that have multiple components and are ultimately aiming to transform healthcare to become learning systems generating and utilising cycles of data more effectively to drive change [17, 24, 27]. Additionally, Patients and carers’ participation depended on many factors such as familiarity with digital tools or preferences and assumptions about the right way to give feedback (e.g. verbal feedback). These findings also resonate with previous research highlighting technical barriers but also how digital interventions can be perceived to threaten established meaning or valued healthcare reletionships if they are expected to replace or change patient-professional relationships [7, 12, 28].

Equally, collective action was variable and only a few staff felt they needed to change their roles and responsibilities. A small number of staff acknowledged that digital feedback would reduce their work, especially with regard to analysing data, but many saw it as an added burden or as another mandatory task. The technical hitches that occurred regularly meant that patients did not always maintain their faith in the equipment. Staff did not feel responsible for sorting technical problems. Others have drawn attention to the barriers that emerge when shifting burdens of work related tasks alongside implementation of complex remote care interventions [30]. The data here demonstrate similar challenges are evident even when patients and staff are together in a shared space and when seemingly small tasks are required to implement a change, and this has implications for the wider agenda to promote greater digital engagement within routine health care settings [42].

It was too early to systematically collect data on reflexive monitoring but there were indications that staff could see the benefits of embedding the new system as their routine option, particularly in site C2. While benefits were identified in the other sites no discernible behaviour change took place, and particularly in site B structural barriers were highlighted.

In a recent paper May and colleagues [43] put forward a theoretical expansion of NPT by considering it in relation to understanding context as a process. They draw on the concept of complex adaptive systems that means these systems consist of different participants and components that are dynamic, interactive and dependent. By doing so, NPT constructs are related to context in a way that allows the implementation process to be seen as non-linear, and a result of a series of feedback loops, negotiations and emergent restructuring. In this paper we have demonstrated an extension of this lense on complex adaptive systems in application and comparison across four distinct organisational settings. Applying these additional insights to the DEPEND project means that the lack of collective action (no stability in staff involved), normative rules about what constitutes core work (eg, consultations focus on clinical issues rather than explaining feedback systems; or reception staff not considering guidance about the kiosk as part of their job) and perceived lack of workability (unreliable technology that needs regular monitoring) created a context that did not allow for consistent implementation.

Considering the context as process that was continually changed and adapted by the perceptions and actions of participants aids understanding the potentially contradictory forces in the implementation of a new complex intervention such as the digital feedback methods from DEPEND. The differences between the sites demonstrate this: in the acute site initial commitment appeared strong as digital feedback was considered to make organisational sense, but at the individual level workability was perceived in variable ways and no consistent pattern of implementation emerged. Our analysis showed that the teams on the ground felt quite distant from the organisational and managerial level that had historically taken ownership of the processes of collecting and interpreting patient experience data. The two primary care sites differed markedly mainly because the leadership perceived their role in contrasting ways with one practice expecting the research team to drive the process, and their involvement was intermittent and inconsistent. In the other practice a senior GP and the practice manager felt that driving the new feedback approach forward was part of their remit, and redefined the core work of the PPG with its members through continuous debate. This ‘looping backwards and forwards’ took place throughout the life of the project, demonstrating the point that the context could be considered as a process. The continuing cycle of discussion and adaptation meant that the implementation process was tailored to the specific context and thus allowed digital feedback to be implemented and commitment to future follow-up was made.

As outlined above, NPT points to organisational complexity as having a major bearing on the likelihood of successful adoption of healthcare technologies (see also, Greenhalgh et al., 21) and here the most complex site was the mental health trust. The senior managers considered that digital feedback had much potential for the organisation, and grassroots staff were generally positive about testing a new approach of eliciting and recording verbal feedback via the care coordinator. Yet, the major structural reorganisation of the Trust impacted on collective action as staff changes meant that initial cognitive participation was not carried over. The instability and insecurity following the organisational change also had a negative effect on workability as many staff felt overburdened with adapting to new organisational processes and work related to the digital feedback project was seen to add to their burden instead of alleviating it. Thus, contradictions between the perceived value of the intervention and its enactment within a stressed environment shaped a context whereby forces favourable and unfavourable to implementation were in constant flux. Consequently, no clear route to establishing digital feedback could be created.

The NPT findings are summarised in Table 2. The wider contextual changes that have shaped the implementation and routinisation are referred to where relevant.

**Table 2:** Summary of results using the Normalization process theory coding framework

Coherence	Cognitive participation	Collective action	Reflexive monitoring
**Differentiation:** Staff in all sites understood that the digital feedback system differed from existing practice. Patients/social networks also saw this difference.	**Enrolment:** Buy-in from senior staff existed but this was not necessarily the case amongst staff who had to implement or support the change.	**Skill set workability:** Most staff did not feel that their roles and responsibilities were affected.	**Reconfiguration:** Spatial changes were made in several sites to make the kiosk more visible. Alternatives to the keyboard were also introduced.
**Communal specification:** Shared understanding amongst staff varied between sites. Senior staff and data analysts had greatest understanding.	**Activation:** Continued involvement was strongest in sites A and C2.	**Contextual Integration:** Organizational support was highest in site A and C2. The context in site C2 for continuous feedback loops and PPG involvement strengthened implementation. Initial support in site C1 was adversely affected by the major organisational changes.	**Communal appraisal:** Potential benefits were recognised, but the study was not able to collect longer term views.
**Individual specification:** Agreement about tasks and responsibilities in the implementation of the new approach was variable across sites with most clear agreement in site C2.	**Initiation:** Site 2 had a lead GP and practice manager as key drivers. Site A had senior management support but grassroots staff did not always feel connected. In site B initial commitment was high but organisational change and turbulence reduced this.	**Interactional workability:** Staff with data analysis responsibilities felt that the new approach would ease their work. Grassroots staff were concerned that supporting patients to use the digital kiosk would add to their workload.	**Individual appraisal;** Lasting effects on individuals and their work environment are not yet discernible.
**Internalization:** The potential value of digital feedback was understood by staff, patients/social networks.	**Legitimation:** Staff differed in their belief that supporting digital feedback was part of their role. Patients/social networks were at times hampered by their lack of confidence to engage with digital tools.	**Relational integration:** Staff confidence in the new system was adversely affected by technical hitches. Patients’ confidence depended on their own digital skills and physical ability to operate the keyboard.	**Systematization:** Benefits in terms of quicker data analysis are recognised. Longer term benefits may be identified in the future.

## Conclusions

Adopting a theory-based framework such as the NPT augmented with a more in-depth consideration of context through the concept of complex adaptive systems has allowed the analysis of the implementation of digital feedback to be more dynamic and to take account of the multiple actors involved in formulating and actioning the work required. By using four different case study sites the barriers, contradictions and drivers for change in organisational practices could be better understood. Patient and carer perspectives were equally more nuanced by taking into account the different personal and structural factors that shaped their perspectives on the usefulness of digital tools. Analysing the diversity of perspectives within context provides insights into how interventions should be targeted and tailored to specific needs. Continual adaptation to changing circumstances is integral to ensuring that an intervention remains relevant and thus embedded in daily practice. The findings raise a number of implications for practice including the need for positive leadership, appropriate information and support. The comparison across multiple sites demonstrated that having a staff champion for innovation, as well as appropriate information and peer support were most supportive for enabling change within these complex adaptive systems.

## Data Availability

The datasets generated and/or analysed during the current study are not publicly available due to conditions of ethics approval and but are available from the corresponding author on reasonable request.

## Abbreviations

FFT: Friends and Family Test
NPT: Normalisation Process Theory
DEPEND: Developing and Enhancing the Usefulness of Patient Experience and Narrative Data
FG: Focus Group
CMHT: Community Mental Health Team
OPT: Outpatients Department
GP: General Practitioner
PPGs: Patient Participation Groups

## Footnotes

1 ^1^ The text mining programmes were developed and tested using retrospective patient experience data Available for download via the following link http://gnteam.cs.manchester.ac.uk/depend/

## References

[1] Campbell J, Smith P, Nissen S, Bower P, Elliott M, Roland M (2009). The GP patient survey for use in primary care in the National Health Service in the UK - development and psychometric characteristics. BMC Fam Pract.

[2] NHS England Insight Team (2014). Review of the friends and family test – inpatient and accident and emergency settings.

[3] Francis R (2013). Report of the mid Staffordshire NHS Foundation trust public inquiry.

[4] Berwick D (2013). A promise to learn – a commitment to act: improving the safety of patients in England.

[5] NHS England. The NHS long term plan. London; 2019. https://www.longtermplan.nhs.uk/wp-content/uploads/2019/08/nhs-long-term-plan-version-1.2.pdf. Accessed 26 Apr 2020.

[6] Scoble S, Schlepper L (2018). Digital patients: myth and reality.

[7] Murray E, Hekler EB, Andersson G, Collins LM, Doherty A, Hollis C, Rivera DE, West R, Wyatt JC (2016). Evaluating digital health interventions: key questions & approaches. Am J Prev Med.

[8] Morton K, Dennison L, May C, Murray E, Little P, McManus RJ, Yardley L (2017). Using digital interevntions for self-management of chronic physical health conditions: a meta-ethnography review of published studies. Patient Educ Couns.

[9] Neves Barbosa B, Waycott J, Malta S. Old and afraid of new communication technologies? Reconceptualising and contesting the ‘age-based digital divide’. J Sociol. 2018:1–13. 10.1177/1440783318766119.

[10] Powell J, Boylan A-M, Greaves F (2015). Harnessing patient feddback data: a challenge for policy and service. Editorial Digit Health.

[11] Latif A, Waring J, Pollock K, Solomon J, Gulzar N, Choudary S, Anderson C (2019). Towards equity: a qualitative exploration of the implementation and impact of a digital educational intervention for pharmacy professionals in England. Int J Equity Health.

[12] Sanders C, Rogers A, Bowen R, Bower P, Hirani S, Cartwright M, Fitzpatrick R, Knapp M, Barlow J, Hendy J, Chrysanthaki T, Bardsley M, Newman SP (2012). Exploring barriers to participation and adoption of telehealth and telecare within the whole system demonstrator trial: a qualitative study. BMC Health Serv Res.

[13] Neves Barbosa B, Amaro F, Fonseca J (2013). Coming of (old) age in the digital age: ICT usage and non-usage among older adults. Soc Res Online.

[14] Coulter A, Locock L, Ziebland S, Calabrese J (2014). Collecting data on patient experience is not enough. BMJ.

[15] Desai A, Zoccatelli G, Adams M, Allen D, Brearley S, Rafferty AM, Donnetto S (2017). Taking data seriously: the value of actor-network theory in rethinking patient experience data. J Health Serv Res Pol.

[16] Martin PG, McKee L, Dixon-Woods M (2015). Beyond metrics? Utilizing ‘soft intelligence’ for healthcare quality and safety. Soc Sci Med.

[17] Gude WT, Roos-Blom M-J, van der Veer S, Dongelmans DA, de Jonge E, Francis J, Peek N, de Keizer NF (2018). Health professionals’ perceptions about their clinical performance and the influence of audit and feedback on their intentions to improve practice: a theory-based study in Dutch intensive care units. Implement Sci.

[18] Moore G, Audrey S, Barker M, Bond L, Bonell C, Hardeman W, Moore L, O’Cathain A, Tinati T, Wight D, Baird J (2015). Process evaluation of complex interventions: Medical Research Council guidance. BMJ.

[19] Damschroder L, Aron D, Keith R, Kirsh S, Alexander J, Lowery J (2009). Fostering implementation of health services research findings into practice: a consolidated framework for advancing implementation science. Impl Sci.

[20] Greenhalgh T, Robert G, Macfarlane F, Bate P, Kyriakidou O (2004). Diffusion of innovations in service organizations: systematic review and recommendations. Milbank Q.

[21] Greenhalgh T, Wherton J, Papoutsi C, Lynch J, Hughes G, A’Court C, Hinder S, Fahy N, Proctor R, Shaw S (2017). Beyond adoption: A new framework for theorizing and evaluating nonadoption, abandonment, and challenges to the scale-up, spread and sustainability of health and care technologies. J Med Internet Res.

[22] May C, Mair F, Finch T, McFarlane A, Dowrick C, Treweek S, Rapley T, Ballini L, Ong BN, Rogers A, Murray E, Elwyn G, Légaré F, Gunn J, Montori V (2009). Development of a theory of implementation and integration: normalization process theory. Impl Sci.

[23] Ross J, Stevenson F, Dack C, Pal K, May C, Michie S, Barnard M, Murray E (2018). Developing an implementation strategy for a digital health intervention: an example in routine healthcare. BMC Health Serv Res.

[24] Gude WT, Roos-Blom M-J, van der Veer S, Dongelmans DA, de Jonge E, Peek N, de Keizer NF (2019). Facilitating action planning within audit and feedback interventions: a mixed methods process evaluation of an action implementation toolbox in intensive care. Impl Sci.

[25] Vest JR, Jung HY, Wiley K, Kooreman MA, Pettit L, Unruh MA (2019). Adoption of health information technology among US nursing facilities. J Am Med Dir Assoc.

[26] Chen PT, Lin CL, Wu WN. Big data management in healthcare: adoption challenges and implications. Int J Inf Manage. 2020. 10.1016/j.ijinfomgt.2020.102078.

[27] Kujala S, Horhammer I, Heponiemi T, Josefsson K (2019). The role of frontline leaders in building health professional support for a new patient portal: survey study. JMIR.

[28] Tsai JCN, Hung SY (2016). Determinants of knowledge management system adoption in health care. J Org Comp Elect Com.

[29] Jeffries M, Keers RN, Phipps DL, Williams R, Brown B, Avery AJ, Peek N, Ashcroft DM (2018). Developing a learning health system: insights from a qualitative process evaluation of a pharmacist-led electronic audit and feedback intervention to improve medication safety in primary care. PLoS One.

[30] Mair F, May C (2012). O’Donnell, Finch T, Sullivan F, Murray E. factors that promote or inhibit the implementation of e-health systems: an explanatory systematic reviews. Bull WHO.

[31] May C, Finch T (2009). Implementing, embedding and integrating practices: an outline of normalization process theory. Sociology..

[32] May C (2013). Towards a general theory of implementation. Implementation Sci.

[33] McEvoy R, Ballini L, Maltoni S, O’Donnell CA, Mair FS, MacFarlane A (2014). A qualitative systematic review of studies using the normalization process theory to research implementation processes. Impl Sci.

[34] May C, Cummings A, Girling M, Bracher M, Mair FS, May CM, Murray E, Myall M, Rapley T, Finch T (2018). Using normalization process theory in feasibility studies and process evaluations of complex healthcare interventions: a systematic review. Impl Sci.

[35] Sanders C, Nenadic G, Dixon W, Lewis S, Boaden R, Bower P et al. Enhancing the credibility, usefulness and relevance of patient experience data in services for people with long-term physical and mental health conditions using digital data capture and improved analysis of narrative data (DEPEND). Study Protocol. https://www.journalslibrary.nihr.ac.uk/programmes/hsdr/1415616/#/. Accessed 10 Apr 2020.

[36] Ong BN, Sanders C. Exploring digital engagement with digital screens for collecting patient feedback in clinical waiting rooms – the role of touch and place. Health. 2019. 10.1177/1363459319889097. 10.1177/1363459319889097 PMC8276334 31814439

[37] Patton M (2009). Qualitative research and evaluation methods.

[38] Strauss A, Corbin J (2009). The discovery of grounded theory.

[39] Charmaz K (2006). Constructing grounded theory. A practical guide through qualitative analysis.

[40] McNaughton R, Steven A, Shucksmith J. Using Normalization Process Theory as a practical tool across the life course of a qualitative research project. Qual Health Res. 2019:1–10. 10.1177/1049732319863420. 10.1177/1049732319863420 31347440

[41] Robert G, Cornwell J, Black N (2018). Friends and family test should no longer be mandatory. BMJ.

[42] Honeyman M, Dunn P, & McKenna H. An introduction to the digital agenda and plans for implementation. The King’s Fund. 2016. https: //www.kingsfund.org.uk/sites/default/files/field/field_publication_file/A_digital_NHS_Kings_Fund_Sep_2016.pdf. Accessed 10 Apr 2020.

[43] May C, Johnson M, Finch T (2016). Implementation, context and complexity. Impl Sci.

